# Synthesis and crystal structure of ebastinium hydrogen fumarate

**DOI:** 10.1107/S2056989022008118

**Published:** 2022-08-18

**Authors:** Prabhakar Priyanka, Bidarur K. Jayanna, Haruvegowda Kiran Kumar, Vinaya, Thayamma R. Divakara, Hemmige S. Yathirajan, Sean Parkin

**Affiliations:** a Department of Chemistry, B. N. M. Institute of Technology, Bengaluru-560 070, India; b Department of Studies in Chemistry, University of Mysore, Manasagangotri, Mysuru-570 006, India; c T. John Institute of Technology, Bengaluru-560 083, India; d Department of Chemistry, University of Kentucky, Lexington, KY, 40506-0055, USA; University of Aberdeen, Scotland

**Keywords:** crystal structure, ebastine, hydrogen fumarate, pseudo-merohedral twinning, disorder

## Abstract

The structure of ebastinium hydrogen fumarate {systematic name: 1-[4-(4-*tert*-butyl­phen­yl)-4-oxobut­yl]-4-(di­phenyl­meth­oxy)piperidin-1-ium (*E*)-3-carb­oxy-1-hy­droxy­prop-2-en-1-olate}, C_32_H_40_NO_2_^+^·C_4_H_3_O_4_^−^, a 1:1 salt formed in the reaction between ebastine and fumaric acid is presented. All examined crystals were found to be twinned by pseudo-merohedry. The structure is extensively disordered, with over half (20 out of 35) its non-hydrogen atoms modelled as lying over two sets of sites. In the crystal, cation–anion pairs are linked by a strong N—H⋯O hydrogen bond [N⋯O = 2.697 (11) Å]. These units inter­act *via* weaker C—H⋯O and C—H⋯π contacts to form layers lying parallel to the *bc* plane. The hydrogen fumarate anions are linked by a very short O—H⋯O hydrogen bond [O⋯O = 2.5402 (17) Å], augmented by weak C—H⋯O contacts into pairs of *R* ^2^_2_(6) ring motifs to form chains that extend parallel to the *b*-axis direction. Comparisons to similar crystal structures are presented.

## Abstract 2

Crystals of the title salt are twinned by pseudo-merohedry and the structure shows extensive disorder. A strong N—H⋯O hydrogen bond links the cation and the anion and the anions are linked into [010] chains by O—H⋯O hydrogen bonds.

## 1. Chemical context

The second-generation anti­histamine ebastine, C_32_H_39_NO_2_, systematic name 4-(benzyl­hydroxy)-1-{4-[4-(tert-but­yl)phen­yl]-4-oxobut­yl}piperidine, is an H_1_ receptor antagonist that acts by blocking H_1_ receptors *via* its carb­oxy­lic acid metabolite, carebastine (Yamaguchi *et al.*, 1994 ▸). It is prescribed mainly for allergic rhinitis and chronic idiopathic urticaria (hives) (Van Cauwenberge *et al.*, 2004 ▸). A review of its pharmacological properties and clinical efficacy in the treatment of allergic disorders has been reported by Wiseman & Faulds (1996 ▸). Formulations of ebastine and its salts with various counter-anions have been the subject of numerous patents (see, for example, Bobee *et al.*, 1995 ▸; Roma-Millan *et al.*, 2011 ▸; Bilgic, 2013 ▸). In spite of this, only the crystal structures of the neutral free-base mol­ecule (Cheng *et al.*, 2005 ▸; Sharma *et al.*, 2015 ▸) and the salt ebastinium 3,5-di­nitro­benzoate (Shaibah *et al.*, 2017 ▸) have been reported to date. By contrast, fumarates (di-anion fumarate and mono-anion hydrogen fumarate) are common counter-anions in compounds of pharmacological importance; examples include opipramolium fumarate (Siddegowda *et al.*, 2011 ▸), cinnarizinium fumarate (Kavitha *et al.*, 2013 ▸) (technically, both hydrogen fumarates), and the recently reported structure of bis­(4-acet­oxy-*N*,*N*-di­methyl­tryptammonium)­fumarate, a new crystalline form of psilacetin (Chadeayne *et al.*, 2019 ▸). As part of our studies in this area, we now report the synthesis and crystal structure of the title 1:1 salt ebastinium hydrogen fumarate, C_32_H_40_NO_2_^+^·C_4_H_3_O_4_^−^, (**I**), formed in the reaction between ebastine and fumaric acid. 
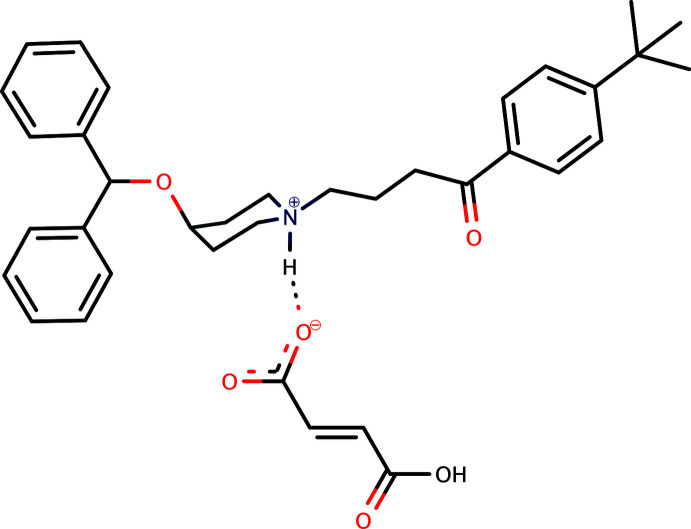


## 2. Structural commentary

**Figure 1:**
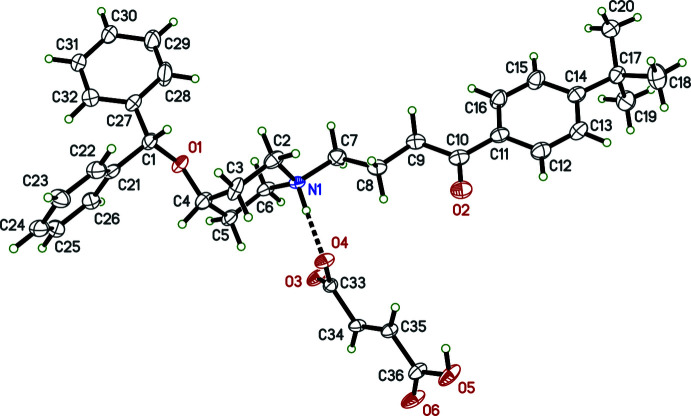
An ellipsoid plot (50% probability) of **I**. The N—H⋯O hydrogen bond is shown as a dashed line. The minor component of disorder is omitted to enhance clarity.

**Figure 2:**
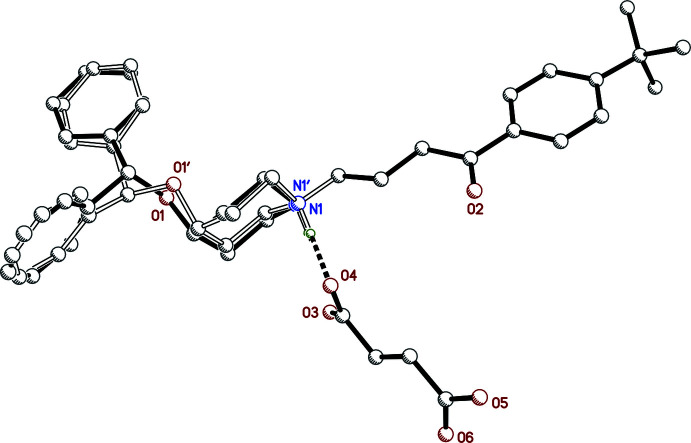
A ball-and-stick plot showing the superposition of major (solid bonds) and minor (open bonds) in ebastinium hydrogen fumarate, **I**. Hydrogen atoms (except for piperidinium NH) are omitted to enhance clarity.

All examined samples of **I** were twinned by pseudo-merohedry, as is common for monoclinic crystals with *β* close to 90° (see, for example, Parkin, 2021 ▸). Further details on how this was handled are given in section 6 (*Crystal handling, data collection and structure refinement*). The asymmetric unit of **I** (Fig. 1 ▸) consists of a single ebastinium cation–hydrogen fumarate anion pair. The cation is extensively disordered, with over half (20 out of 35) its non-H atoms modelled as occupying two sets of sites, with refined occupancy factors of 0.729 (4) and 0.271 (4), as shown in Fig. 2 ▸. Unless stated otherwise, the numerical details in the following description apply to the major conformation.

The ebastinium cation is protonated at N1 (Fig. 1 ▸), which in turn forms a strong N—H⋯O hydrogen bond to the carboxyl­ate O4 atom of the hydrogen fumarate anion [N1⋯O4 = 2.697 (11) Å, Table 1 ▸]. The piperidinium ring of the cation is in the expected chair conformation, with the 4-*t-*butyl­phenyl-4-oxobutyl substituent equatorial at N1 and the di­phenyl­meth­oxy substituent axial at C4, similar to the salt described by Shaibah *et al.* (2017 ▸), who also noted that this axial substitution is in contrast to the equatorial placement in free-base ebastine (Cheng *et al.*, 2005 ▸; Sharma *et al.*, 2015 ▸). The phenyl-4-oxobutyl fragment is largely planar (r.m.s. deviation = 0.0814 Å for atoms C7–C16 and O2): the main deviation [0.1879 (13) Å] is at atom C7, as seen in the C7—C8—C9—C10 torsion angle of −168.02 (14)°. The major and minor disorder conformations arise as a result of superposition of components that differ primarily by rotation of the di­phenyl­meth­oxy group about the C4—O1 and C1—O1 bonds (Fig. 2 ▸), the torsion angles C1—O1—C4—C5 and C4—O1—C1—C27 being 177.4 (3) and 175.6 (3)°, respectively, for the major disorder component compared to 85.8 (11) and 68.67 (11)°, respectively, for the minor component. The dihedral angle between the phenyl rings is 73.41 (18)° in the major component [*c.f.* 73.3 (6)°, minor]. Additional details concerning the disorder are given in section 6 (*Crystal handling, data collection and structure refinement*).

The hydrogen fumarate anion deviates substanti­ally from planarity, as indicated by the dihedral angle between its carboxyl­ate and carb­oxy­lic acid groups of 23.51 (14)°. As expected, the C—O bond lengths in the deprotonated carboxyl­ate group [1.2638 (18) and 1.2503 (18) Å for C33—O3 and C33—O4, respectively] are the same within the accuracy limitations of the spherical-atom scattering-factor approximation (see, for example, Dawson, 1964 ▸), while those of the carb­oxy­lic acid group [1.3197 (19) and 1.211 (2) Å for C36—O5 and C36—O6, respectively] are significantly different. Indeed, throughout the whole structure there are no unusual bond lengths or angles in either species.

## 3. Supra­molecular features

For the sake of clarity, the following description is restricted to the major component of disorder except where stated otherwise. The packing in **I** features only two types of conventional hydrogen bonds; the strong N1—H1*N*⋯O4 [N⋯O = 2.697 (11) Å, Table 1 ▸] link and a very short [2.5402 (17) Å] O5—H5*O*⋯O3^iii^ hydrogen bond between hydrogen fumarate anions (*vide infra*). Much weaker C—H⋯O hydrogen bonds connect the ebastinium cations along the *b*-axis direction (C7—H7*A*⋯O2^i^), ebastinium and hydrogen fumarate ions *via* the *c*-glide (C8—H8*B*⋯O6^ii^) and hydrogen fumarate anions into chains parallel to the *b*-axis direction (C34—H34⋯O5^i^ and C35—H35⋯O3^iii^). The symmetry operations are those defined in the footnote to Table 1 ▸. Since these weaker inter­actions do not involve disordered atoms, the above description applies equally well to both major and minor components. There are no aromatic π–π stacking inter­actions, but there are C—H⋯π close contacts between the phenyl rings of the disordered di­phenyl­meth­oxy group, which are also summarized in Table 1 ▸.

**Figure 3:**
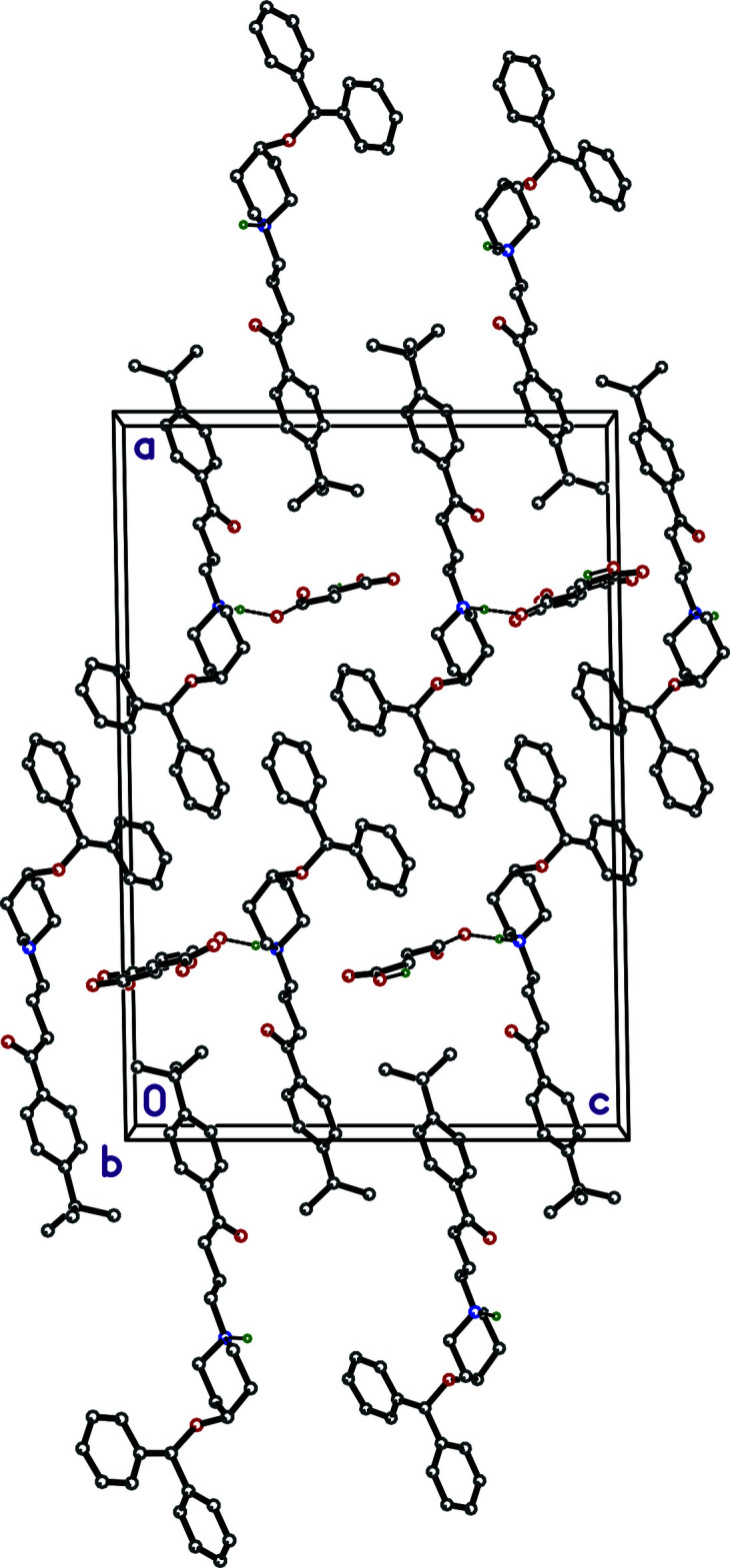
A packing plot of **I** viewed down the *b*-axis direction showing how the 4-*t-*butyl phenyl groups inter­digitate with *c*-glide related copies of themselves, leading to layers that extend parallel to the *bc*-plane centered on *a* = 0 (and 1), and stack along the *a*-axis direction. The strong hydrogen bonds between cation and anion (*i.e.*, N1—H1*N*⋯O4) are shown as thick dashed lines.

**Figure 4:**
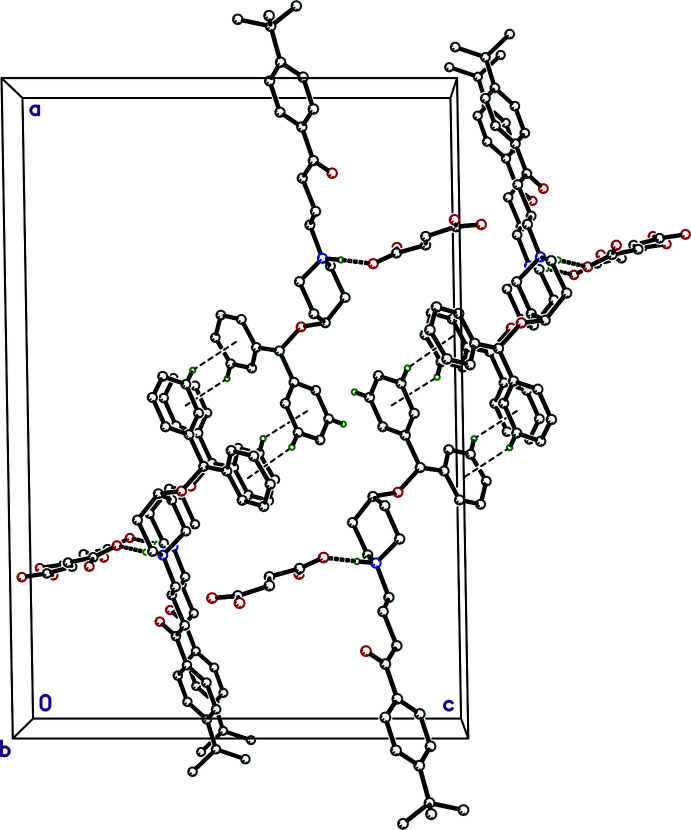
A partial packing plot of **I** viewed down the *b*-axis direction showing C—H⋯π contacts (thin dashed lines) between the di­phenyl­meth­oxy groups, thereby forming the inter­face, centered on *a* = 1/2, between layers parallel to the *bc* plane. In the inter­ests of clarity, the minor component of disorder is suppressed.

**Figure 5:**
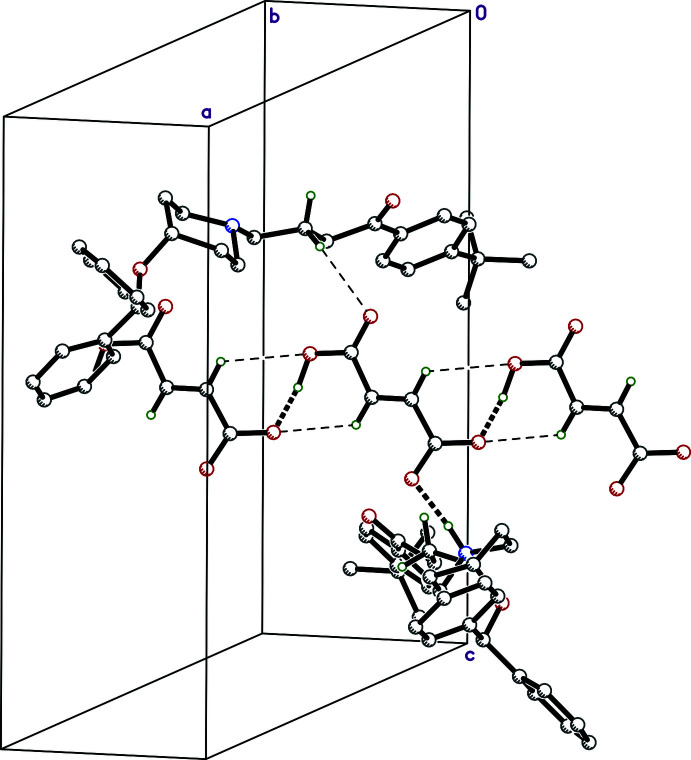
A partial packing plot showing three hydrogen fumarate anions connected into a chain running horizontally (parallel to *b*) and the contacts of the central anion with ebastinium cations. Strong hydrogen bonds (O—H⋯O, N—H⋯O) are shown as thick dashed lines whereas weak C—H⋯O hydrogen bonds are shown as thin dashed lines. Pairs of 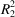 (6) ring motifs illustrate how the weak C—H⋯O inter­actions augment the strong O—H⋯O hydrogen bond.

The main structural motif in the extended structure of **I** is the cation–anion pair (Fig. 1 ▸). In the crystal, chemically distinct groups are segregated such that the 4-*t-*butyl­phenyl groups inter­digitate with *c*-glide-related copies of themselves (Fig. 3 ▸) and the di­phenyl­meth­oxy groups inter­act *via* the aforementioned C—H⋯π contacts (Fig. 4 ▸), forming layers that extend parallel to the *bc* plane and stack along the *a*-axis direction. The hydrogen fumarate anions form chains that propagate along the *b-*axis direction by virtue of the O5—H5*O*⋯O3^iii^, C34—H34⋯O5^i^ and C35—H35⋯O3^iii^ hydrogen bonds (Fig. 5 ▸), which form pairs of 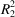 (6) ring motifs (Etter *et al.*, 1990 ▸).

**Figure 6:**
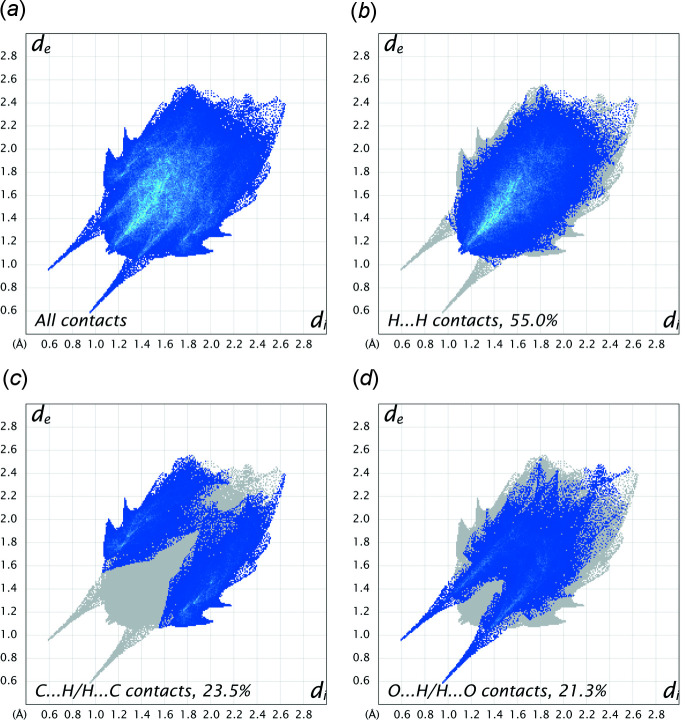
Fingerprint plots of inter­atomic contacts for **I** (major disorder component only) obtained from a Hirshfeld surface analysis. (*a*) All contacts, (*b*) H⋯H contacts (55% coverage), (*c*) C⋯H/H⋯C contacts (23.5%), (*d*) O⋯H/H⋯O contacts (21.3%).

A rigorous Hirshfeld surface analysis (Spackman & Jayatilaka, 2009 ▸) is complicated by the extensive disorder in **I**, but fingerprint plots generated for the major disorder component using *CrystalExplorer* (Spackman *et al.*, 2021 ▸) (Fig. 6 ▸) provide a reasonable summary of atom–atom contacts (Fig. 6 ▸). The most prevalent are H⋯H contacts (55%), followed by C⋯H/H⋯C (23.5%) and O⋯H/H⋯O (21.3%), with all other contacts being negligible.

## 4. Database survey

A search of the Cambridge Structure Database (version 5.43 with updates as of June 2022; Groom *et al.*, 2016 ▸) for the keywords ‘ebastine’ or ‘ebastinium’ revealed only two hits, CSD refcode QATJIF (Cheng *et al.*, 2005 ▸) and the duplicate QATJIF01 (Sharma *et al.*, 2015 ▸); both are structures of the free base, ebastine. An ebastinium salt with 3,5-di­nitro­benzoate was not returned in this search, but is present as entry HECMIO (Shaibah *et al.*, 2017 ▸). A search using the mol­ecular fragment that extends from the ether oxygen atom through to and including the benzene ring (atoms O1/O2/N1/C2–C16 in this report) but including no other atoms, gave 38 unique structures (46 hits, eight of which were duplicates). Many of these were originally published in the pharmaceutical chemistry literature, highlighting the medicinal importance of the central core of the ebastine mol­ecule. In contrast, a search for the keyword ‘fumarate’ gave 434 hits, covering a wide variety of structures with both the mono-anion and di-anion.

**Figure 7:**
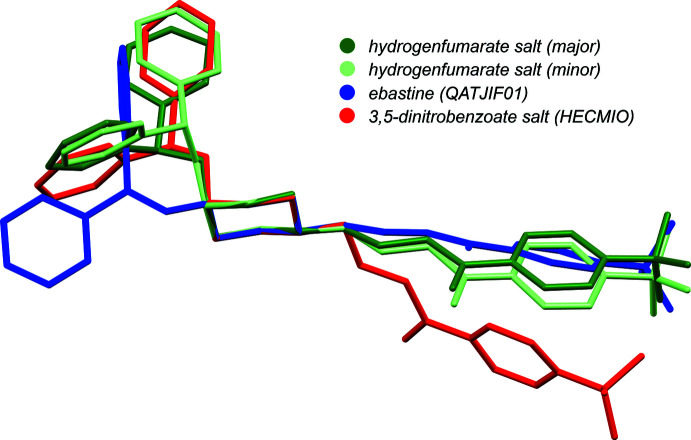
An overlay of the major and minor conformations of the ebastinium cation in **I** (this work) with ebastine (CSD: QATJIF01) and ebastinium cation from the 3,5-di­nitro­benzoate salt (CSD: HECMIO, major conformation only), from a least-squares fit of non-H atoms in the piperidine/piperidinium rings. The axial placement of the di­phenyl­meth­oxy group (at left) in the salts is clearly different from the equatorial placement of the free base (blue). For the sake of clarity, only the major disorder component of HECMIO is shown. Diagram generated using *Mercury* (Macrae *et al.*, 2020 ▸).

A detailed comparison of the ebastine structure (coord­inates taken from QATJIF01) with the 3,5-di­nitro­benzoate salt (HECMIO) was made by Shaibah *et al.* (2017 ▸). The free base (*i.e*., QATJIF and QATJIF01) is not disordered, but HECMIO has a relatively simple two-component disorder of the benzene ring of its 4-*t-*butyl­phenyl substituent. Of partic­ular note (Shaibah *et al.*, 2017 ▸) was the placement of the (C_6_H_5_)_2_CHO group relative to the piperidine/piperidinium ring, which is equatorial in ebastine, but axial in the ebastinium salt. The (C_6_H_5_)_2_CHO substituent in both disorder components of the hydrogen fumarate salt presented here is axial, as in HECMIO. The conformation of the C_4_H_6_O-4-*t-*butyl­phenyl fragment in **I**, however, is more similar to that in the neutral mol­ecule (QATJIF and QATJIF01). An overlay of the major and minor disorder components of **I** with QATJIF01 and HECMIO highlights these conformational differences (Fig. 7 ▸).

## 5. Synthesis, crystallization and spectroscopic details

A sample of ebastine was obtained as a gift from R. L. Fine Chemicals, Bengaluru, India. Ebastine (100 mg, 0.21 mmol) and fumaric acid (25 mg, 0.21 mmol) were dissolved in hot ethyl acetate and DMF and stirred over a heating magnetic stirrer for 30 minutes at 333 K. The resulting solution was allowed to cool slowly to room temperature with slow evaporation. Crystallization was carried out using several solvents (ethyl acetate/DMF, acetone, aceto­nitrile, and methyl­ethyl ketone) *via* slow evaporation to give plate-shaped crystals in about a week (m.p. 468–470 K). All crystals observed were twinned by pseudo-merohedry, but those grown from aceto­nitrile were the largest and gave the best diffraction patterns [see section 6 (*Crystal handling, data collection and structure refinement*) for further details].

NMR spectra were recorded on an SA-AGILENT 400MHz NMR spectrometer: ^1^H NMR: DMSO-*d*_6_ (400 MHz, δ ppm): 1.294 [*s*, 9H, C—(CH_3_)_3_]; 1.615–1.592 (*d*, 2H, *J* = 9.2 Hz, CH_2_); 1.871–1.818 (*q*, 4H, *J* = 6.8 Hz, piperidine); 2.400 (*b*, 2H, O=C—CH_2_); 2.576–2.541 (*t*, 2H, *J* = 7.2 Hz, piperidine); 2.870 (*s*, 2H, piperidine); 3.024–2.989 (*t*, 2H, *J* = 6.8 Hz, CH_2_); 3.423–3.406 (*b*, 1H, –CH), 5.63 (*s*, 1H, –CH); 6.557 (*s*, 2H, HC=CH); 7.250–7.207 (*m*, 2H, phen­yl); 7.372–7.297 (*m*, 8H, phen­yl); 7.533–7.512 (*d*, 2H, *J* = 8.4 Hz, phen­yl); 7.891–7.870 (*d*, 2H, *J* = 8.4 Hz, phen­yl); 11.6–14.2 (*b*, 1H, OH). ^13^C NMR: DMSO-*d*_6_ (100 MHz, δ ppm): 20.04, 29.53, 30.78, 34.76, 35.25, 49.52, 55.93, 79.06, 125.39, 126.57, 127.13, 127.80, 128.25, 134.15, 134.57, 142.96, 156.03, 167.03, 198.80.

## 6. Crystal handling, data collection and structure refinement

Crystals from each of the aforementioned solvents [see section 5 (*Synthesis, crystallization and spectroscopic details*)] were thin plates that indexed to give essentially the same unit-cell dimensions. All specimens were pseudo-merohedral twins by virtue of the *β* angle being close to 90° and had roughly equal component volume fractions, as determined by the refined BASF parameter in *SHELXL* (Sheldrick, 2015*b* ▸) for the twin operation corresponding to 180° rotation about the *c*-axis. A small number of the crystals grown from aceto­nitrile were somewhat thicker than most specimens, such that it was possible to cut along the twin plane, thereby separating individuals. Data collected from such a separated thin slice gave better refinement statistics than any of the uncut crystals. Nevertheless, the twin model was retained for the final refinement because, in spite of the very low occupancy minor component fraction of 0.19 (2)%, its standard uncertainty is only about one tenth as large, and is therefore of statistical significance. Even with such a tiny residual minor individual fraction, refinement statistics were marginally better with TWIN/BASF (in *SHELXL*) included. For a concise description of the various types of twinning that commonly affect mol­ecular crystals, particularly twinning by pseudo-merohedry and the attendant twin operations that constitute the twin law, see Parkin (2021 ▸).

In addition to the twinning, the structure is extensively disordered. This disorder consists of a rotation of the (C_6_H_5_)_2_CHO group of the cation followed by a relaxation into the available space, which in turn places the whole of the (C_6_H_5_)_2_CHO group in two distinct orientations [see section 2 (*Structural commentary*)]. This of necessity must also cause minor site splitting of the piperidinium ring, albeit not discernible in electron-density maps calculated to 0.77 Å resolution. The two largest difference map peaks are only about 0.5 and 0.4 electrons, but are in positions that suggest a third, much smaller, disorder component. Such an additional disorder component, however, was not modelled due to its necessarily minuscule occupancy fraction. To ensure satisfactory refinement for disordered atom sequences in the structure, a combination of restraints were employed. The *SHELXL* commands SAME and SADI were used to maintain the chemical integrity and similarity of the disordered groups, while RIGU and SIMU were used to ensure physically reasonable displacement parameters for closely proximate disordered atom pairs.

Crystal data, data collection and structure refinement details are summarized in Table 2 ▸. All non-disordered and major-component H atoms were found in difference-Fourier maps. Carbon-bound hydrogen atoms were subsequently included in the refinement using riding models, with constrained distances set to 0.95 Å (*R*_2_C*sp* ^2^H), 0.98 Å (*R*CH_3_), 0.99 Å (*R*_2_CH_2_) and 1.00 Å (*R*_3_CH). The N—H hydrogen atom was included using a riding model that allowed the N—H distance to refine, while that of the minor component was constrained. The O—H hydrogen atom coordinates of the hydrogen fumarate anion were refined freely. *U*_iso_(H) parameters were set to values of either 1.2*U*_eq_ (*R*_2_C_ar_H, *R*_2_CH_2_, *R*_3_CH, NH) or 1.5*U*_eq_ (*R*CH_3_, OH) of the attached atom. The final structure was checked using validation tools in *PLATON* (Spek, 2020 ▸) and *checkCIF*.

## Supplementary Material

Crystal structure: contains datablock(s) global, I. DOI: 10.1107/S2056989022008118/hb8035sup1.cif

Structure factors: contains datablock(s) I. DOI: 10.1107/S2056989022008118/hb8035Isup2.hkl

Supporting information file. DOI: 10.1107/S2056989022008118/hb8035Isup3.cml

CCDC reference: 2201292

Additional supporting information:  crystallographic information; 3D view; checkCIF report

## Figures and Tables

**Table 1:** Hydrogen-bond geometry (Å, °) *Cg*1 and *Cg*2 represent the centroids of phenyl rings C21–C26 and C27–C32, respectively.

*D*—H⋯*A*	*D*—H	H⋯*A*	*D*⋯*A*	*D*—H⋯*A*
N1—H1*N*⋯O4	0.95	1.75	2.697 (11)	175
N1′—H1*N*′⋯O4	1.00	1.78	2.77 (3)	169
O5—H5*O*⋯O3^i^	1.04 (2)	1.50 (2)	2.5402 (17)	171 (2)
C7—H7*A*⋯O2^ii^	0.99	2.37	3.330 (2)	164
C8—H8*B*⋯O6^iii^	0.99	2.53	3.325 (2)	137
C34—H34⋯O5^ii^	0.95	2.62	3.208 (2)	121
C35—H35⋯O3^i^	0.95	2.49	3.1450 (19)	127
C31—H31⋯*Cg*1^iv^	0.95	2.72	3.534 (6)	145
C25—H25⋯*Cg*1^v^	0.95	2.70	3.532 (4)	146
C23—H23⋯*Cg*2^vi^	0.95	2.75	3.624 (4)	154

Symmetry codes: (i) 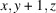 ; (ii) 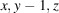 ; (iii) 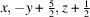 ; (iv) 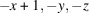 ; (v) 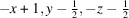 ; (vi) 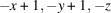 .

**Table 2:** Experimental details

Crystal data	
Chemical formula	C_32_H_40_NO_2_^+^·C_4_H_3_O_4_^−^
*M*_r_	585.71
Crystal system, space group	Monoclinic, *P*2_1_/*c*
Temperature (K)	90
*a*, *b*, *c* (Å)	27.091 (3), 6.2408 (5), 18.685 (2)
β (°)	90.975 (3)
*V* (Å^3^)	3158.6 (6)
*Z*	4
Radiation type	Mo *K*α
μ (mm^−1^)	0.08
Crystal size (mm)	0.24 × 0.14 × 0.03

Data collection	
Diffractometer	Bruker D8 Venture dual source
Absorption correction	Multi-scan (*SADABS*; Krause *et al.*, 2015 ▸)
*T*_min_, *T*_max_	0.857, 0.959
No. of measured, independent and observed [*I* > 2σ(*I*)] reflections	55813, 7224, 5164
*R*_int_	0.047
(sin θ/λ)_max_ (Å^−1^)	0.649

Refinement	
*R*[*F* ^2^ > 2σ(*F* ^2^)], *wR*(*F* ^2^), *S*	0.047, 0.114, 1.03
No. of reflections	7224
No. of parameters	578
No. of restraints	445
H-atom treatment	H atoms treated by a mixture of independent and constrained refinement
Δρ_max_, Δρ_min_ (e Å^−3^)	0.52, −0.19

Computer programs: *APEX3* (Bruker, 2016 ▸), *SHELXT* (Sheldrick, 2015*a* ▸), *SHELXL2019/2* (Sheldrick, 2015*b* ▸), *XP* in *SHELXTL* (Sheldrick, 2008 ▸), *SHELXTL* (Sheldrick, 2008 ▸) and *publCIF* (Westrip, 2010 ▸).

## supplementary crystallographic information

### Crystal data

**Table:**

C_32_H_40_NO_2_^+^·C_4_H_3_O_4_^−^	*F*(000) = 1256
*M**_r_* = 585.71	*D*_x_ = 1.232 Mg m^−^^3^
Monoclinic, *P*2_1_/*c*	Mo *K*α radiation, λ = 0.71073 Å
*a* = 27.091 (3) Å	Cell parameters from 9606 reflections
*b* = 6.2408 (5) Å	θ = 2.3–27.3°
*c* = 18.685 (2) Å	µ = 0.08 mm^−^^1^
β = 90.975 (3)°	*T* = 90 K
*V* = 3158.6 (6) Å^3^	Plate, colourless
*Z* = 4	0.24 × 0.14 × 0.03 mm

### Data collection

**Table:**

Bruker D8 Venture dual source diffractometer	7224 independent reflections
Radiation source: microsource	5164 reflections with *I* > 2σ(*I*)
Detector resolution: 7.41 pixels mm^-1^	*R*_int_ = 0.047
φ and ω scans	θ_max_ = 27.5°, θ_min_ = 2.2°
Absorption correction: multi-scan (*SADABS*; Krause *et al.*, 2015)	*h* = −35→35
*T*_min_ = 0.857, *T*_max_ = 0.959	*k* = −7→8
55813 measured reflections	*l* = −24→24

### Refinement

**Table:**

Refinement on *F*^2^	Secondary atom site location: difference Fourier map
Least-squares matrix: full	Hydrogen site location: mixed
*R*[*F*^2^ > 2σ(*F*^2^)] = 0.047	H atoms treated by a mixture of independent and constrained refinement
*wR*(*F*^2^) = 0.114	*w* = 1/[σ^2^(*F*_o_^2^) + (0.0333*P*)^2^ + 1.537*P*] where *P* = (*F*_o_^2^ + 2*F*_c_^2^)/3
*S* = 1.03	(Δ/σ)_max_ = 0.001
7224 reflections	Δρ_max_ = 0.52 e Å^−^^3^
578 parameters	Δρ_min_ = −0.19 e Å^−^^3^
445 restraints	Extinction correction: SHELXL2019/2 (Sheldrick 2015b), Fc^*^=kFc[1+0.001xFc^2^λ^3^/sin(2θ)]^-1/4^
Primary atom site location: structure-invariant direct methods	Extinction coefficient: 0.0017 (4)

### Special details

**Table:**

Experimental. The crystal was mounted using polyisobutene oil on the tip of a fine glass fibre, which was fastened in a copper mounting pin with electrical solder. It was placed directly into the cold gas stream of a liquid-nitrogen based cryostat (Hope, 1994; Parkin & Hope, 1998). Diffraction data were collected with the crystal at 90K, which is standard practice in this laboratory for the majority of flash-cooled crystals.
Geometry. All esds (except the esd in the dihedral angle between two l.s. planes) are estimated using the full covariance matrix. The cell esds are taken into account individually in the estimation of esds in distances, angles and torsion angles; correlations between esds in cell parameters are only used when they are defined by crystal symmetry. An approximate (isotropic) treatment of cell esds is used for estimating esds involving l.s. planes.
Refinement. Refined as a 2-component twin.

### Fractional atomic coordinates and isotropic or equivalent isotropic displacement parameters (Å^2^)

**Table:**

	*x*	*y*	*z*	*U*_iso_*/*U*_eq_	Occ. (<1)
C7	0.20849 (6)	0.7170 (3)	−0.16752 (10)	0.0296 (4)	
H7A	0.185362	0.615042	−0.190991	0.036*	
H7B	0.208016	0.689703	−0.115336	0.036*	
C8	0.19117 (6)	0.9435 (3)	−0.18209 (9)	0.0274 (4)	
H8A	0.190854	0.969760	−0.234348	0.033*	
H8B	0.214680	1.045616	−0.159546	0.033*	
C9	0.14002 (6)	0.9833 (3)	−0.15337 (9)	0.0276 (4)	
H9A	0.118220	0.862657	−0.167570	0.033*	
H9B	0.141905	0.986853	−0.100427	0.033*	
C10	0.11752 (6)	1.1900 (3)	−0.18015 (9)	0.0271 (4)	
C11	0.06723 (6)	1.2518 (3)	−0.15588 (9)	0.0261 (4)	
C12	0.04494 (7)	1.4361 (3)	−0.18329 (10)	0.0337 (4)	
H12	0.061969	1.520883	−0.217124	0.040*	
C13	−0.00150 (7)	1.4973 (3)	−0.16206 (10)	0.0347 (4)	
H13	−0.015849	1.623305	−0.181951	0.042*	
C14	−0.02801 (6)	1.3794 (3)	−0.11218 (9)	0.0279 (4)	
C15	−0.00537 (6)	1.1957 (3)	−0.08535 (11)	0.0347 (4)	
H15	−0.022282	1.111001	−0.051361	0.042*	
C16	0.04111 (6)	1.1326 (3)	−0.1067 (1)	0.0339 (4)	
H16	0.055326	1.005624	−0.087336	0.041*	
C17	−0.08065 (6)	1.4408 (3)	−0.09209 (10)	0.0296 (4)	
C18	−0.08751 (8)	1.6832 (3)	−0.09150 (13)	0.0486 (5)	
H18A	−0.082349	1.740047	−0.139684	0.073*	
H18B	−0.063553	1.747785	−0.058064	0.073*	
H18C	−0.121055	1.717576	−0.076368	0.073*	
C19	−0.11537 (7)	1.3419 (3)	−0.14877 (11)	0.0415 (5)	
H19A	−0.107029	1.397234	−0.196135	0.062*	
H19B	−0.149580	1.379546	−0.138061	0.062*	
H19C	−0.111696	1.185656	−0.148334	0.062*	
C20	−0.09453 (7)	1.3543 (3)	−0.01864 (10)	0.0426 (5)	
H20A	−0.127537	1.405422	−0.006443	0.064*	
H20B	−0.070520	1.404689	0.017402	0.064*	
H20C	−0.094425	1.197298	−0.019745	0.064*	
O2	0.13952 (4)	1.30433 (19)	−0.22189 (7)	0.0338 (3)	
N1	0.2608 (3)	0.6772 (14)	−0.1950 (5)	0.0207 (10)	0.729 (4)
H1N	0.2646 (3)	0.758 (3)	−0.2376 (12)	0.025*	0.729 (4)
O1	0.36765 (7)	0.3639 (4)	−0.14095 (11)	0.0329 (5)	0.729 (4)
C1	0.40693 (8)	0.4053 (5)	−0.08943 (14)	0.0318 (6)	0.729 (4)
H1	0.401014	0.548461	−0.067124	0.038*	0.729 (4)
C2	0.2990 (2)	0.7535 (8)	−0.1414 (4)	0.0299 (10)	0.729 (4)
H2A	0.297565	0.664873	−0.097570	0.036*	0.729 (4)
H2B	0.292141	0.904014	−0.128130	0.036*	0.729 (4)
C3	0.34996 (19)	0.7376 (6)	−0.1732 (3)	0.0337 (9)	0.729 (4)
H3A	0.374828	0.782540	−0.136773	0.040*	0.729 (4)
H3B	0.352040	0.837638	−0.214153	0.040*	0.729 (4)
C4	0.3623 (2)	0.5110 (6)	−0.1987 (2)	0.0319 (10)	0.729 (4)
H4	0.393500	0.514544	−0.226516	0.038*	0.729 (4)
C5	0.3207 (2)	0.4260 (11)	−0.2459 (3)	0.0288 (9)	0.729 (4)
H5A	0.327102	0.273168	−0.256541	0.035*	0.729 (4)
H5B	0.320539	0.504963	−0.291875	0.035*	0.729 (4)
C6	0.2701 (3)	0.4459 (13)	−0.2127 (4)	0.0251 (10)	0.729 (4)
H6A	0.244433	0.393366	−0.246661	0.030*	0.729 (4)
H6B	0.268802	0.358040	−0.168659	0.030*	0.729 (4)
C21	0.45774 (19)	0.4068 (10)	−0.1223 (3)	0.0292 (10)	0.729 (4)
C22	0.49210 (17)	0.5609 (7)	−0.1025 (2)	0.0336 (9)	0.729 (4)
H22	0.483326	0.665914	−0.068179	0.040*	0.729 (4)
C23	0.53888 (16)	0.5668 (6)	−0.1312 (2)	0.0350 (9)	0.729 (4)
H23	0.561954	0.673858	−0.116928	0.042*	0.729 (4)
C24	0.55123 (14)	0.4129 (7)	−0.18122 (19)	0.0336 (9)	0.729 (4)
H24	0.583134	0.413880	−0.201546	0.040*	0.729 (4)
C25	0.51727 (14)	0.2575 (6)	−0.20178 (19)	0.0327 (8)	0.729 (4)
H25	0.526049	0.152763	−0.236204	0.039*	0.729 (4)
C26	0.47069 (15)	0.2537 (8)	−0.1725 (3)	0.0306 (9)	0.729 (4)
H26	0.447625	0.146595	−0.186809	0.037*	0.729 (4)
C27	0.40034 (17)	0.2352 (7)	−0.0330 (2)	0.0351 (10)	0.729 (4)
C28	0.3544 (2)	0.2194 (9)	0.0017 (3)	0.0645 (16)	0.729 (4)
H28	0.328738	0.316813	−0.010989	0.077*	0.729 (4)
C29	0.3459 (2)	0.0678 (7)	0.0528 (3)	0.0559 (15)	0.729 (4)
H29	0.315266	0.065145	0.076755	0.067*	0.729 (4)
C30	0.3820 (3)	−0.0819 (13)	0.0699 (5)	0.0431 (11)	0.729 (4)
H30	0.376530	−0.184382	0.106513	0.052*	0.729 (4)
C31	0.4256 (2)	−0.0818 (12)	0.0338 (4)	0.0336 (12)	0.729 (4)
H31	0.450309	−0.185406	0.044800	0.040*	0.729 (4)
C32	0.4336 (2)	0.0710 (8)	−0.0191 (3)	0.0347 (12)	0.729 (4)
H32	0.462801	0.062460	−0.046450	0.042*	0.729 (4)
N1'	0.2574 (7)	0.701 (4)	−0.1860 (14)	0.022 (2)	0.271 (4)
H1N'	0.259455	0.772729	−0.233740	0.026*	0.271 (4)
O1'	0.3683 (2)	0.4743 (11)	−0.1213 (3)	0.0396 (15)	0.271 (4)
C1'	0.4022 (2)	0.2951 (12)	−0.1234 (4)	0.0342 (15)	0.271 (4)
H1'	0.391924	0.195831	−0.162870	0.041*	0.271 (4)
C2'	0.2961 (6)	0.808 (2)	−0.1404 (12)	0.030 (2)	0.271 (4)
H2'1	0.298622	0.734598	−0.093513	0.036*	0.271 (4)
H2'2	0.286471	0.958811	−0.131678	0.036*	0.271 (4)
C3'	0.3462 (5)	0.8030 (17)	−0.1763 (9)	0.0317 (19)	0.271 (4)
H3'1	0.344118	0.882281	−0.222156	0.038*	0.271 (4)
H3'2	0.371053	0.874878	−0.145181	0.038*	0.271 (4)
C4'	0.3621 (5)	0.5761 (19)	−0.1902 (7)	0.031 (2)	0.271 (4)
H4'	0.393715	0.573293	−0.217142	0.037*	0.271 (4)
C5'	0.3218 (6)	0.452 (3)	−0.2305 (9)	0.032 (2)	0.271 (4)
H5'1	0.320513	0.501750	−0.280718	0.039*	0.271 (4)
H5'2	0.330949	0.298078	−0.230940	0.039*	0.271 (4)
C6'	0.2710 (7)	0.473 (4)	−0.1995 (12)	0.024 (2)	0.271 (4)
H6'1	0.246455	0.408576	−0.233113	0.029*	0.271 (4)
H6'2	0.269747	0.391902	−0.154000	0.029*	0.271 (4)
C21'	0.3942 (5)	0.182 (2)	−0.0496 (6)	0.035 (2)	0.271 (4)
C22'	0.3642 (7)	0.278 (3)	0.0050 (9)	0.067 (3)	0.271 (4)
H22'	0.352840	0.422012	0.000961	0.080*	0.271 (4)
C23'	0.3527 (6)	0.152 (2)	0.0638 (9)	0.057 (3)	0.271 (4)
H23'	0.329001	0.198259	0.097343	0.068*	0.271 (4)
C24'	0.3762 (9)	−0.040 (4)	0.0722 (14)	0.044 (2)	0.271 (4)
H24'	0.366492	−0.136032	0.108873	0.053*	0.271 (4)
C25'	0.4143 (6)	−0.096 (4)	0.0273 (11)	0.033 (3)	0.271 (4)
H25'	0.432588	−0.222590	0.037098	0.040*	0.271 (4)
C26'	0.4264 (6)	0.026 (2)	−0.0308 (9)	0.037 (3)	0.271 (4)
H26'	0.455824	0.001951	−0.056525	0.044*	0.271 (4)
C27'	0.4542 (5)	0.368 (3)	−0.1355 (9)	0.028 (2)	0.271 (4)
C28'	0.4738 (4)	0.546 (2)	−0.0998 (8)	0.037 (3)	0.271 (4)
H28'	0.453933	0.622240	−0.067147	0.045*	0.271 (4)
C29'	0.5210 (4)	0.6117 (17)	−0.1108 (6)	0.038 (2)	0.271 (4)
H29'	0.533659	0.733207	−0.085975	0.046*	0.271 (4)
C30'	0.5502 (4)	0.5038 (19)	−0.1574 (7)	0.040 (3)	0.271 (4)
H30'	0.582673	0.556029	−0.164371	0.048*	0.271 (4)
C31'	0.5355 (4)	0.3231 (19)	−0.1952 (6)	0.041 (3)	0.271 (4)
H31'	0.556234	0.246170	−0.226675	0.049*	0.271 (4)
C32'	0.4869 (4)	0.268 (2)	−0.1814 (7)	0.033 (3)	0.271 (4)
H32'	0.474349	0.146602	−0.206531	0.040*	0.271 (4)
C33	0.25492 (5)	0.8367 (2)	−0.36524 (8)	0.0206 (3)	
C34	0.24432 (6)	0.9666 (2)	−0.43083 (8)	0.0223 (3)	
H34	0.244724	0.896818	−0.476010	0.027*	
C35	0.23444 (5)	1.1729 (2)	−0.42912 (9)	0.0214 (3)	
H35	0.235790	1.247094	−0.384736	0.026*	
C36	0.22119 (6)	1.2914 (3)	−0.49577 (9)	0.0261 (4)	
O3	0.24297 (4)	0.64104 (17)	−0.36785 (6)	0.0304 (3)	
O4	0.27305 (4)	0.92704 (17)	−0.31116 (6)	0.0265 (3)	
O5	0.21455 (5)	1.49991 (18)	−0.48923 (7)	0.0369 (3)	
H5O	0.2245 (8)	1.545 (3)	−0.4372 (13)	0.055*	
O6	0.21637 (5)	1.20367 (19)	−0.55327 (7)	0.0395 (3)	

### Atomic displacement parameters (Å^2^)

**Table:**

	*U*^11^	*U*^22^	*U*^33^	*U*^12^	*U*^13^	*U*^23^
C7	0.0280 (8)	0.0308 (9)	0.0302 (10)	−0.0044 (7)	0.0025 (7)	0.0052 (8)
C8	0.0312 (8)	0.0279 (9)	0.0230 (9)	−0.0066 (7)	−0.0002 (7)	−0.0013 (7)
C9	0.0299 (8)	0.0301 (9)	0.0229 (9)	−0.0060 (7)	0.0007 (7)	0.0008 (7)
C10	0.0329 (8)	0.0271 (9)	0.0212 (9)	−0.0090 (7)	−0.0032 (7)	−0.0015 (7)
C11	0.0303 (8)	0.0265 (9)	0.0215 (9)	−0.0061 (7)	−0.0029 (7)	0.0008 (7)
C12	0.0403 (10)	0.0300 (9)	0.0308 (10)	−0.0033 (8)	0.0016 (8)	0.0061 (8)
C13	0.0404 (10)	0.0324 (10)	0.0313 (11)	0.0038 (8)	−0.0028 (8)	0.0095 (8)
C14	0.0298 (8)	0.0295 (9)	0.0241 (9)	−0.0034 (7)	−0.0063 (7)	−0.0004 (7)
C15	0.0318 (9)	0.0326 (10)	0.0399 (12)	−0.0005 (7)	0.0027 (8)	0.0135 (8)
C16	0.0323 (9)	0.031 (1)	0.0384 (11)	0.0021 (7)	0.0007 (8)	0.0112 (8)
C17	0.0307 (8)	0.0285 (9)	0.0296 (10)	0.0017 (7)	−0.0040 (7)	0.0015 (7)
C18	0.0464 (11)	0.0335 (11)	0.0659 (16)	0.0049 (9)	0.0049 (10)	−0.0018 (10)
C19	0.0307 (9)	0.0531 (13)	0.0405 (12)	0.0018 (9)	−0.0076 (8)	−0.0038 (10)
C20	0.0396 (10)	0.0557 (13)	0.0325 (12)	0.0125 (9)	0.0025 (8)	0.0068 (9)
O2	0.0381 (7)	0.0319 (7)	0.0315 (7)	−0.0075 (5)	0.0038 (5)	0.0050 (6)
N1	0.0245 (14)	0.025 (2)	0.013 (2)	−0.0017 (12)	−0.0006 (14)	0.0005 (14)
O1	0.0351 (10)	0.0387 (13)	0.0246 (11)	0.0098 (10)	−0.0080 (8)	0.0074 (9)
C1	0.0331 (12)	0.0414 (15)	0.0206 (13)	0.0147 (11)	−0.007 (1)	−0.0027 (11)
C2	0.0399 (15)	0.026 (3)	0.0240 (14)	−0.0039 (17)	−0.0106 (12)	0.0003 (19)
C3	0.0280 (14)	0.029 (2)	0.0435 (17)	0.0007 (17)	−0.0142 (12)	0.010 (2)
C4	0.0319 (13)	0.035 (2)	0.0285 (17)	0.0042 (17)	−0.0026 (12)	0.0098 (16)
C5	0.0330 (13)	0.033 (2)	0.020 (2)	0.0066 (13)	−0.0064 (14)	0.0012 (15)
C6	0.0378 (14)	0.021 (2)	0.016 (3)	−0.0012 (13)	−0.0046 (15)	0.0018 (15)
C21	0.0361 (15)	0.035 (2)	0.016 (2)	0.0113 (14)	−0.0043 (14)	0.0032 (15)
C22	0.040 (3)	0.0371 (18)	0.0242 (16)	0.0061 (19)	0.002 (2)	0.0003 (13)
C23	0.041 (2)	0.037 (2)	0.027 (2)	0.0045 (18)	0.0052 (16)	−0.0052 (16)
C24	0.0412 (17)	0.037 (3)	0.023 (2)	0.0087 (17)	0.0037 (14)	0.0009 (17)
C25	0.0326 (19)	0.041 (2)	0.0247 (15)	0.0082 (15)	0.0017 (15)	−0.0042 (14)
C26	0.0297 (19)	0.0383 (18)	0.0238 (19)	0.0035 (16)	−0.0013 (15)	−0.0022 (14)
C27	0.0293 (16)	0.053 (2)	0.0228 (18)	0.0100 (16)	−0.0067 (14)	0.0046 (15)
C28	0.032 (2)	0.092 (4)	0.070 (2)	0.028 (2)	0.0181 (18)	0.054 (3)
C29	0.0366 (19)	0.079 (3)	0.053 (3)	0.022 (2)	0.0138 (18)	0.037 (3)
C30	0.037 (3)	0.058 (3)	0.0338 (17)	0.0190 (17)	0.0023 (15)	0.017 (2)
C31	0.030 (3)	0.0417 (18)	0.0287 (18)	0.011 (2)	−0.0021 (19)	0.0040 (15)
C32	0.032 (2)	0.042 (2)	0.029 (2)	0.0086 (17)	0.0010 (16)	0.0007 (17)
N1'	0.032 (3)	0.022 (4)	0.011 (5)	0.001 (3)	0.004 (3)	0.005 (3)
O1'	0.049 (3)	0.040 (3)	0.030 (3)	0.015 (3)	−0.003 (2)	0.006 (2)
C1'	0.034 (3)	0.041 (3)	0.028 (3)	0.011 (2)	−0.008 (2)	0.005 (2)
C2'	0.038 (3)	0.027 (5)	0.025 (3)	−0.001 (3)	−0.013 (3)	0.001 (4)
C3'	0.029 (3)	0.026 (4)	0.040 (3)	0.002 (3)	−0.009 (3)	0.013 (4)
C4'	0.029 (3)	0.037 (4)	0.026 (3)	0.002 (3)	−0.007 (3)	0.011 (3)
C5'	0.037 (3)	0.035 (4)	0.025 (5)	0.010 (3)	−0.011 (3)	0.002 (3)
C6'	0.033 (3)	0.024 (4)	0.016 (5)	0.001 (3)	−0.009 (3)	0.001 (3)
C21'	0.025 (3)	0.053 (4)	0.027 (4)	0.004 (3)	−0.009 (3)	0.015 (3)
C22'	0.048 (5)	0.093 (5)	0.060 (4)	0.037 (4)	0.017 (4)	0.047 (4)
C23'	0.041 (4)	0.077 (5)	0.054 (4)	0.030 (4)	0.013 (4)	0.030 (4)
C24'	0.030 (4)	0.066 (5)	0.036 (3)	0.017 (3)	0.006 (3)	0.017 (4)
C25'	0.022 (5)	0.044 (4)	0.032 (4)	0.005 (4)	−0.003 (4)	0.006 (3)
C26'	0.034 (4)	0.047 (5)	0.030 (4)	0.007 (4)	−0.003 (3)	0.004 (4)
C27'	0.032 (3)	0.033 (4)	0.019 (4)	0.006 (3)	−0.007 (3)	0.004 (3)
C28'	0.030 (5)	0.047 (5)	0.035 (5)	0.003 (4)	−0.004 (4)	0.000 (4)
C29'	0.027 (5)	0.044 (5)	0.044 (6)	0.001 (4)	−0.003 (4)	−0.013 (4)
C30'	0.037 (4)	0.039 (7)	0.043 (8)	0.005 (4)	0.001 (4)	0.003 (4)
C31'	0.047 (6)	0.042 (7)	0.034 (6)	−0.004 (4)	0.006 (5)	0.002 (4)
C32'	0.039 (6)	0.035 (4)	0.026 (5)	0.005 (4)	0.004 (4)	0.002 (3)
C33	0.0261 (7)	0.0185 (8)	0.0173 (8)	0.0033 (6)	−0.0003 (6)	0.0002 (6)
C34	0.0317 (8)	0.0215 (8)	0.0139 (8)	−0.0015 (6)	0.0011 (6)	−0.0002 (6)
C35	0.0277 (8)	0.0193 (8)	0.0171 (8)	−0.0023 (6)	−0.0025 (6)	0.0001 (6)
C36	0.0379 (9)	0.0186 (8)	0.0215 (9)	−0.0014 (7)	−0.0060 (7)	0.0023 (7)
O3	0.0533 (7)	0.0144 (6)	0.0231 (7)	−0.0002 (5)	−0.0098 (5)	0.0028 (5)
O4	0.0385 (6)	0.0231 (6)	0.0179 (6)	−0.0037 (5)	−0.0042 (5)	0.0009 (5)
O5	0.0676 (9)	0.0156 (6)	0.0268 (7)	0.0021 (6)	−0.0186 (6)	0.0010 (5)
O6	0.0769 (9)	0.0233 (6)	0.0179 (7)	0.0010 (6)	−0.0113 (6)	0.0000 (5)

### Geometric parameters (Å, º)

**Table:**

C7—N1'	1.38 (2)	C25—H25	0.9500
C7—C8	1.513 (2)	C26—H26	0.9500
C7—N1	1.537 (8)	C27—C32	1.387 (6)
C7—H7A	0.9900	C27—C28	1.417 (6)
C7—H7B	0.9900	C28—C29	1.367 (6)
C8—C9	1.515 (2)	C28—H28	0.9500
C8—H8A	0.9900	C29—C30	1.385 (6)
C8—H8B	0.9900	C29—H29	0.9500
C9—C10	1.509 (2)	C30—C31	1.371 (7)
C9—H9A	0.9900	C30—H30	0.9500
C9—H9B	0.9900	C31—C32	1.392 (6)
C10—O2	1.220 (2)	C31—H31	0.9500
C10—C11	1.494 (2)	C32—H32	0.9500
C11—C16	1.386 (2)	N1'—C6'	1.494 (14)
C11—C12	1.392 (2)	N1'—C2'	1.498 (14)
C12—C13	1.380 (2)	N1'—H1N'	1.0000
C12—H12	0.9500	O1'—C4'	1.443 (11)
C13—C14	1.396 (2)	O1'—C1'	1.448 (9)
C13—H13	0.9500	C1'—C27'	1.500 (13)
C14—C15	1.389 (2)	C1'—C21'	1.568 (12)
C14—C17	1.530 (2)	C1'—H1'	1.0000
C15—C16	1.385 (2)	C2'—C3'	1.523 (13)
C15—H15	0.9500	C2'—H2'1	0.9900
C16—H16	0.9500	C2'—H2'2	0.9900
C17—C18	1.524 (3)	C3'—C4'	1.504 (10)
C17—C20	1.528 (3)	C3'—H3'1	0.9900
C17—C19	1.534 (2)	C3'—H3'2	0.9900
C18—H18A	0.9800	C4'—C5'	1.529 (12)
C18—H18B	0.9800	C4'—H4'	1.0000
C18—H18C	0.9800	C5'—C6'	1.508 (14)
C19—H19A	0.9800	C5'—H5'1	0.9900
C19—H19B	0.9800	C5'—H5'2	0.9900
C19—H19C	0.9800	C6'—H6'1	0.9900
C20—H20A	0.9800	C6'—H6'2	0.9900
C20—H20B	0.9800	C21'—C26'	1.350 (15)
C20—H20C	0.9800	C21'—C22'	1.445 (16)
N1—C6	1.503 (5)	C22'—C23'	1.392 (15)
N1—C2	1.505 (6)	C22'—H22'	0.9500
N1—H1N	0.95 (2)	C23'—C24'	1.367 (15)
O1—C4	1.422 (4)	C23'—H23'	0.9500
O1—C1	1.446 (3)	C24'—C25'	1.383 (16)
C1—C27	1.509 (5)	C24'—H24'	0.9500
C1—C21	1.517 (5)	C25'—C26'	1.370 (16)
C1—H1	1.0000	C25'—H25'	0.9500
C2—C3	1.515 (5)	C26'—H26'	0.9500
C2—H2A	0.9900	C27'—C28'	1.393 (14)
C2—H2B	0.9900	C27'—C32'	1.394 (14)
C3—C4	1.531 (4)	C28'—C29'	1.362 (10)
C3—H3A	0.9900	C28'—H28'	0.9500
C3—H3B	0.9900	C29'—C30'	1.362 (11)
C4—C5	1.515 (5)	C29'—H29'	0.9500
C4—H4	1.0000	C30'—C31'	1.385 (12)
C5—C6	1.519 (5)	C30'—H30'	0.9500
C5—H5A	0.9900	C31'—C32'	1.391 (11)
C5—H5B	0.9900	C31'—H31'	0.9500
C6—H6A	0.9900	C32'—H32'	0.9500
C6—H6B	0.9900	C33—O4	1.2503 (18)
C21—C22	1.384 (6)	C33—O3	1.2638 (18)
C21—C26	1.390 (6)	C33—C34	1.493 (2)
C22—C23	1.385 (5)	C34—C35	1.316 (2)
C22—H22	0.9500	C34—H34	0.9500
C23—C24	1.385 (4)	C35—C36	1.487 (2)
C23—H23	0.9500	C35—H35	0.9500
C24—C25	1.387 (5)	C36—O6	1.211 (2)
C24—H24	0.9500	C36—O5	1.3197 (19)
C25—C26	1.384 (4)	O5—H5O	1.04 (2)
			
N1'—C7—C8	108.6 (11)	C25—C24—H24	119.8
C8—C7—N1	112.1 (4)	C26—C25—C24	120.5 (4)
C8—C7—H7A	109.2	C26—C25—H25	119.7
N1—C7—H7A	109.2	C24—C25—H25	119.7
C8—C7—H7B	109.2	C25—C26—C21	119.8 (4)
N1—C7—H7B	109.2	C25—C26—H26	120.1
H7A—C7—H7B	107.9	C21—C26—H26	120.1
C7—C8—C9	111.84 (13)	C32—C27—C28	115.9 (5)
C7—C8—H8A	109.2	C32—C27—C1	124.5 (4)
C9—C8—H8A	109.2	C28—C27—C1	118.9 (4)
C7—C8—H8B	109.2	C29—C28—C27	121.8 (5)
C9—C8—H8B	109.2	C29—C28—H28	119.1
H8A—C8—H8B	107.9	C27—C28—H28	119.1
C10—C9—C8	112.92 (14)	C28—C29—C30	120.1 (5)
C10—C9—H9A	109.0	C28—C29—H29	119.9
C8—C9—H9A	109.0	C30—C29—H29	119.9
C10—C9—H9B	109.0	C31—C30—C29	119.8 (6)
C8—C9—H9B	109.0	C31—C30—H30	120.1
H9A—C9—H9B	107.8	C29—C30—H30	120.1
O2—C10—C11	120.08 (16)	C30—C31—C32	119.7 (6)
O2—C10—C9	120.87 (15)	C30—C31—H31	120.2
C11—C10—C9	119.04 (14)	C32—C31—H31	120.2
C16—C11—C12	117.66 (16)	C27—C32—C31	122.0 (5)
C16—C11—C10	122.76 (15)	C27—C32—H32	119.0
C12—C11—C10	119.57 (15)	C31—C32—H32	119.0
C13—C12—C11	120.99 (17)	C7—N1'—C6'	110.6 (17)
C13—C12—H12	119.5	C7—N1'—C2'	119.4 (16)
C11—C12—H12	119.5	C6'—N1'—C2'	110.5 (13)
C12—C13—C14	121.86 (17)	C7—N1'—H1N'	105.0
C12—C13—H13	119.1	C6'—N1'—H1N'	105.0
C14—C13—H13	119.1	C2'—N1'—H1N'	105.0
C15—C14—C13	116.55 (16)	C4'—O1'—C1'	112.3 (7)
C15—C14—C17	121.72 (16)	O1'—C1'—C27'	111.5 (9)
C13—C14—C17	121.60 (15)	O1'—C1'—C21'	103.0 (7)
C16—C15—C14	121.91 (17)	C27'—C1'—C21'	114.5 (9)
C16—C15—H15	119.0	O1'—C1'—H1'	109.2
C14—C15—H15	119.0	C27'—C1'—H1'	109.2
C15—C16—C11	121.02 (16)	C21'—C1'—H1'	109.2
C15—C16—H16	119.5	N1'—C2'—C3'	111.1 (14)
C11—C16—H16	119.5	N1'—C2'—H2'1	109.4
C18—C17—C20	108.19 (17)	C3'—C2'—H2'1	109.4
C18—C17—C14	111.40 (15)	N1'—C2'—H2'2	109.4
C20—C17—C14	112.13 (14)	C3'—C2'—H2'2	109.4
C18—C17—C19	109.32 (16)	H2'1—C2'—H2'2	108.0
C20—C17—C19	108.70 (16)	C4'—C3'—C2'	110.9 (10)
C14—C17—C19	107.04 (15)	C4'—C3'—H3'1	109.5
C17—C18—H18A	109.5	C2'—C3'—H3'1	109.5
C17—C18—H18B	109.5	C4'—C3'—H3'2	109.5
H18A—C18—H18B	109.5	C2'—C3'—H3'2	109.5
C17—C18—H18C	109.5	H3'1—C3'—H3'2	108.0
H18A—C18—H18C	109.5	O1'—C4'—C3'	106.8 (9)
H18B—C18—H18C	109.5	O1'—C4'—C5'	106.6 (11)
C17—C19—H19A	109.5	C3'—C4'—C5'	111.0 (11)
C17—C19—H19B	109.5	O1'—C4'—H4'	110.8
H19A—C19—H19B	109.5	C3'—C4'—H4'	110.8
C17—C19—H19C	109.5	C5'—C4'—H4'	110.8
H19A—C19—H19C	109.5	C6'—C5'—C4'	114.7 (11)
H19B—C19—H19C	109.5	C6'—C5'—H5'1	108.6
C17—C20—H20A	109.5	C4'—C5'—H5'1	108.6
C17—C20—H20B	109.5	C6'—C5'—H5'2	108.6
H20A—C20—H20B	109.5	C4'—C5'—H5'2	108.6
C17—C20—H20C	109.5	H5'1—C5'—H5'2	107.6
H20A—C20—H20C	109.5	N1'—C6'—C5'	112.1 (15)
H20B—C20—H20C	109.5	N1'—C6'—H6'1	109.2
C6—N1—C2	109.6 (5)	C5'—C6'—H6'1	109.2
C6—N1—C7	112.8 (6)	N1'—C6'—H6'2	109.2
C2—N1—C7	110.8 (5)	C5'—C6'—H6'2	109.2
C6—N1—H1N	107.8	H6'1—C6'—H6'2	107.9
C2—N1—H1N	107.8	C26'—C21'—C22'	119.0 (13)
C7—N1—H1N	107.8	C26'—C21'—C1'	117.0 (11)
C4—O1—C1	117.0 (3)	C22'—C21'—C1'	121.5 (11)
O1—C1—C27	104.2 (2)	C23'—C22'—C21'	117.2 (14)
O1—C1—C21	113.2 (3)	C23'—C22'—H22'	121.4
C27—C1—C21	114.0 (3)	C21'—C22'—H22'	121.4
O1—C1—H1	108.4	C24'—C23'—C22'	118.6 (14)
C27—C1—H1	108.4	C24'—C23'—H23'	120.7
C21—C1—H1	108.4	C22'—C23'—H23'	120.7
N1—C2—C3	109.8 (5)	C23'—C24'—C25'	120.1 (17)
N1—C2—H2A	109.7	C23'—C24'—H24'	120.0
C3—C2—H2A	109.7	C25'—C24'—H24'	120.0
N1—C2—H2B	109.7	C26'—C25'—C24'	122.2 (17)
C3—C2—H2B	109.7	C26'—C25'—H25'	118.9
H2A—C2—H2B	108.2	C24'—C25'—H25'	118.9
C2—C3—C4	112.8 (3)	C21'—C26'—C25'	116.3 (15)
C2—C3—H3A	109.0	C21'—C26'—H26'	121.8
C4—C3—H3A	109.0	C25'—C26'—H26'	121.8
C2—C3—H3B	109.0	C28'—C27'—C32'	114.2 (11)
C4—C3—H3B	109.0	C28'—C27'—C1'	121.5 (11)
H3A—C3—H3B	107.8	C32'—C27'—C1'	124.3 (11)
O1—C4—C5	106.3 (4)	C29'—C28'—C27'	121.4 (13)
O1—C4—C3	112.3 (3)	C29'—C28'—H28'	119.3
C5—C4—C3	109.9 (4)	C27'—C28'—H28'	119.3
O1—C4—H4	109.4	C28'—C29'—C30'	120.2 (12)
C5—C4—H4	109.4	C28'—C29'—H29'	119.9
C3—C4—H4	109.4	C30'—C29'—H29'	119.9
C4—C5—C6	113.6 (4)	C29'—C30'—C31'	124.3 (10)
C4—C5—H5A	108.8	C29'—C30'—H30'	117.8
C6—C5—H5A	108.8	C31'—C30'—H30'	117.8
C4—C5—H5B	108.8	C30'—C31'—C32'	111.8 (10)
C6—C5—H5B	108.8	C30'—C31'—H31'	124.1
H5A—C5—H5B	107.7	C32'—C31'—H31'	124.1
N1—C6—C5	108.9 (5)	C31'—C32'—C27'	128.1 (11)
N1—C6—H6A	109.9	C31'—C32'—H32'	116.0
C5—C6—H6A	109.9	C27'—C32'—H32'	116.0
N1—C6—H6B	109.9	O4—C33—O3	124.32 (14)
C5—C6—H6B	109.9	O4—C33—C34	119.06 (14)
H6A—C6—H6B	108.3	O3—C33—C34	116.56 (14)
C22—C21—C26	118.8 (4)	C35—C34—C33	123.21 (15)
C22—C21—C1	120.5 (4)	C35—C34—H34	118.4
C26—C21—C1	120.7 (4)	C33—C34—H34	118.4
C21—C22—C23	122.1 (4)	C34—C35—C36	120.84 (15)
C21—C22—H22	119.0	C34—C35—H35	119.6
C23—C22—H22	119.0	C36—C35—H35	119.6
C24—C23—C22	118.4 (3)	O6—C36—O5	121.01 (15)
C24—C23—H23	120.8	O6—C36—C35	122.62 (15)
C22—C23—H23	120.8	O5—C36—C35	116.37 (14)
C23—C24—C25	120.4 (4)	C36—O5—H5O	108.7 (12)
C23—C24—H24	119.8		
			
N1'—C7—C8—C9	−171.8 (10)	O1—C1—C27—C28	−56.4 (4)
N1—C7—C8—C9	−178.7 (3)	C21—C1—C27—C28	179.7 (4)
C7—C8—C9—C10	−168.02 (14)	C32—C27—C28—C29	8.4 (7)
C8—C9—C10—O2	1.6 (2)	C1—C27—C28—C29	179.5 (5)
C8—C9—C10—C11	−179.52 (14)	C27—C28—C29—C30	−2.9 (9)
O2—C10—C11—C16	−177.72 (17)	C28—C29—C30—C31	−2.0 (12)
C9—C10—C11—C16	3.4 (2)	C29—C30—C31—C32	0.9 (13)
O2—C10—C11—C12	2.4 (2)	C28—C27—C32—C31	−9.5 (8)
C9—C10—C11—C12	−176.43 (16)	C1—C27—C32—C31	180.0 (5)
C16—C11—C12—C13	0.0 (3)	C30—C31—C32—C27	5.1 (10)
C10—C11—C12—C13	179.89 (16)	C8—C7—N1'—C6'	−158.4 (14)
C11—C12—C13—C14	0.4 (3)	C8—C7—N1'—C2'	72 (2)
C12—C13—C14—C15	−0.5 (3)	C4'—O1'—C1'—C27'	68.7 (11)
C12—C13—C14—C17	−176.41 (16)	C4'—O1'—C1'—C21'	−168.0 (8)
C13—C14—C15—C16	0.1 (3)	C7—N1'—C2'—C3'	−170.6 (18)
C17—C14—C15—C16	176.04 (17)	C6'—N1'—C2'—C3'	60 (2)
C14—C15—C16—C11	0.3 (3)	N1'—C2'—C3'—C4'	−59.2 (19)
C12—C11—C16—C15	−0.4 (3)	C1'—O1'—C4'—C3'	−155.5 (9)
C10—C11—C16—C15	179.75 (16)	C1'—O1'—C4'—C5'	85.8 (11)
C15—C14—C17—C18	148.79 (18)	C2'—C3'—C4'—O1'	−63.7 (15)
C13—C14—C17—C18	−35.5 (2)	C2'—C3'—C4'—C5'	52.2 (16)
C15—C14—C17—C20	27.4 (2)	O1'—C4'—C5'—C6'	67.7 (18)
C13—C14—C17—C20	−156.94 (17)	C3'—C4'—C5'—C6'	−48 (2)
C15—C14—C17—C19	−91.8 (2)	C7—N1'—C6'—C5'	171.5 (18)
C13—C14—C17—C19	83.9 (2)	C2'—N1'—C6'—C5'	−54 (2)
C8—C7—N1—C6	−153.5 (5)	C4'—C5'—C6'—N1'	49 (2)
C8—C7—N1—C2	83.3 (7)	O1'—C1'—C21'—C26'	−170.1 (11)
C4—O1—C1—C27	175.6 (3)	C27'—C1'—C21'—C26'	−48.9 (15)
C4—O1—C1—C21	−60.0 (4)	O1'—C1'—C21'—C22'	−8.3 (15)
C6—N1—C2—C3	61.7 (8)	C27'—C1'—C21'—C22'	112.9 (15)
C7—N1—C2—C3	−173.2 (5)	C26'—C21'—C22'—C23'	−29 (2)
N1—C2—C3—C4	−56.4 (7)	C1'—C21'—C22'—C23'	170.0 (13)
C1—O1—C4—C5	177.4 (3)	C21'—C22'—C23'—C24'	10 (3)
C1—O1—C4—C3	−62.3 (4)	C22'—C23'—C24'—C25'	7 (4)
C2—C3—C4—O1	−67.9 (6)	C23'—C24'—C25'—C26'	−7 (4)
C2—C3—C4—C5	50.2 (6)	C22'—C21'—C26'—C25'	28 (2)
O1—C4—C5—C6	70.8 (6)	C1'—C21'—C26'—C25'	−169.2 (14)
C3—C4—C5—C6	−51.0 (7)	C24'—C25'—C26'—C21'	−11 (3)
C2—N1—C6—C5	−61.5 (8)	O1'—C1'—C27'—C28'	43.1 (15)
C7—N1—C6—C5	174.6 (6)	C21'—C1'—C27'—C28'	−73.4 (15)
C4—C5—C6—N1	57.3 (8)	O1'—C1'—C27'—C32'	−137.7 (14)
O1—C1—C21—C22	137.7 (4)	C21'—C1'—C27'—C32'	105.9 (16)
C27—C1—C21—C22	−103.5 (5)	C32'—C27'—C28'—C29'	0.6 (15)
O1—C1—C21—C26	−42.5 (6)	C1'—C27'—C28'—C29'	179.9 (15)
C27—C1—C21—C26	76.4 (6)	C27'—C28'—C29'—C30'	−0.1 (14)
C26—C21—C22—C23	−0.1 (7)	C28'—C29'—C30'—C31'	−1.2 (17)
C1—C21—C22—C23	179.8 (5)	C29'—C30'—C31'—C32'	1.7 (17)
C21—C22—C23—C24	0.0 (6)	C30'—C31'—C32'—C27'	−1 (2)
C22—C23—C24—C25	0.1 (5)	C28'—C27'—C32'—C31'	0 (2)
C23—C24—C25—C26	−0.1 (5)	C1'—C27'—C32'—C31'	−179.2 (14)
C24—C25—C26—C21	0.1 (6)	O4—C33—C34—C35	−27.3 (2)
C22—C21—C26—C25	0.0 (7)	O3—C33—C34—C35	150.19 (16)
C1—C21—C26—C25	−179.9 (5)	C33—C34—C35—C36	−176.29 (14)
O1—C1—C27—C32	113.8 (4)	C34—C35—C36—O6	3.9 (3)
C21—C1—C27—C32	−10.1 (5)	C34—C35—C36—O5	−176.43 (15)

### Hydrogen-bond geometry (Å, º)

*Cg*1 and *Cg*2 represent the centroids of phenyl rings C21–C26 and C27–C32, respectively.

**Table:**

*D*—H···*A*	*D*—H	H···*A*	*D*···*A*	*D*—H···*A*
N1—H1*N*···O4	0.95	1.75	2.697 (11)	175
N1′—H1*N*′···O4	1.00	1.78	2.77 (3)	169
O5—H5*O*···O3^i^	1.04 (2)	1.50 (2)	2.5402 (17)	171 (2)
C7—H7*A*···O2^ii^	0.99	2.37	3.330 (2)	164
C8—H8*B*···O6^iii^	0.99	2.53	3.325 (2)	137
C34—H34···O5^ii^	0.95	2.62	3.208 (2)	121
C35—H35···O3^i^	0.95	2.49	3.1450 (19)	127
C31—H31···*Cg*1^iv^	0.95	2.72	3.534 (6)	145
C25—H25···*Cg*1^v^	0.95	2.70	3.532 (4)	146
C23—H23···*Cg*2^vi^	0.95	2.75	3.624 (4)	154

Symmetry codes: (i) *x*, *y*+1, *z*; (ii) *x*, *y*−1, *z*; (iii) *x*, −*y*+5/2, *z*+1/2; (iv) −*x*+1, −*y*, −*z*; (v) −*x*+1, *y*−1/2, −*z*−1/2; (vi) −*x*+1, −*y*+1, −*z*.

## References

[bb1] Bilgic, M. (2013). World Patent number WO-2013/081562.

[bb2] Bobee, J.-M., Conrath, G., Gousset, G., Ponsot, M. & Veillard, M. (1995). US Patent number US-5460829.

[bb3] Bruker (2016). *APEX3*. Bruker AXS Inc., Madison, Wisconsin, USA.

[bb4] Chadeayne, A. R., Golen, J. A. & Manke, D. R. (2019). *Acta Cryst.* E**75**, 900–902. 10.1107/S2056989019007370 PMC6658936 31391991

[bb5] Cheng, J., Zhou, Z. & Yang, G. (2005). *Acta Cryst.* E**61**, o2932–o2933.

[bb6] Dawson, B. (1964). *Acta Cryst.* **17**, 990–996.

[bb7] Etter, M. C., MacDonald, J. C. & Bernstein, J. (1990). *Acta Cryst.* B**46**, 256–262. 10.1107/s0108768189012929 2344397

[bb8] Groom, C. R., Bruno, I. J., Lightfoot, M. P. & Ward, S. C. (2016). *Acta Cryst.* B**72**, 171–179. 10.1107/S2052520616003954 PMC4822653 27048719

[bb9] Kavitha, C. N., Yildirim, S. O., Jasinski, J. P., Yathirajan, H. S. & Butcher, R. J. (2013). *Acta Cryst.* E**69**, o142–o143. 10.1107/S1600536812051239 PMC3588249 23476398

[bb10] Krause, L., Herbst-Irmer, R., Sheldrick, G. M. & Stalke, D. (2015). *J. Appl. Cryst.* **48**, 3–10. 10.1107/S1600576714022985 PMC4453166 26089746

[bb11] Macrae, C. F., Sovago, I., Cottrell, S. J., Galek, P. T. A., McCabe, P., Pidcock, E., Platings, M., Shields, G. P., Stevens, J. S., Towler, M. & Wood, P. A. (2020). *J. Appl. Cryst.* **53**, 226–235. 10.1107/S1600576719014092 PMC6998782 32047413

[bb12] Parkin, S. R. (2021). *Acta Cryst.* E**77**, 452–465. 10.1107/S205698902100342X PMC8100258 34026247

[bb13] Roma-Millan, J., Mestre-Castell, J. & Suñé-Negre, J. M. (2011). European Patent number EP-1944028.

[bb14] Shaibah, M. A. E., Sagar, B. K., Yathirajan, H. S., Kumar, S. M. & Glidewell, C. (2017). *Acta Cryst.* E**73**, 1513–1516. 10.1107/S205698901701324X PMC5730306 29250369

[bb15] Sharma, R., Prasher, D. & Tiwari, R. K. (2015). *J. Appl. Cryst.* **48**, 1299–1301.

[bb16] Sheldrick, G. M. (2008). *Acta Cryst.* A**64**, 112–122. 10.1107/S0108767307043930 18156677

[bb17] Sheldrick, G. M. (2015*a*). *Acta Cryst.* A**71**, 3–8.

[bb18] Sheldrick, G. M. (2015*b*). *Acta Cryst.* C**71**, 3–8.

[bb19] Siddegowda, M. S., Jasinski, J. P., Golen, J. A., Yathirajan, H. S. & Swamy, M. T. (2011). *Acta Cryst.* E**67**, o2296. 10.1107/S160053681103159X PMC3200775 22064675

[bb20] Spackman, M. A. & Jayatilaka, D. (2009). *CrystEngComm*, **11**, 19–32.

[bb21] Spackman, P. R., Turner, M. J., McKinnon, J. J., Wolff, S. K., Grimwood, D. J., Jayatilaka, D. & Spackman, M. A. (2021). *J. Appl. Cryst.* **54**, 1006–1011. 10.1107/S1600576721002910 PMC8202033 34188619

[bb22] Spek, A. L. (2020). *Acta Cryst.* E**76**, 1–11. 10.1107/S2056989019016244 PMC6944088 31921444

[bb23] Van Cauwenberge, P., De Belder, T. & Sys, L. (2004). *Expert Opin. Pharmacother.* **5**, 1807–1813. 10.1517/14656566.5.8.1807 15264995

[bb24] Westrip, S. P. (2010). *J. Appl. Cryst.* **43**, 920–925.

[bb25] Wiseman, L. R. & Faulds, D. (1996). *Drugs*, **51**, 260–277. 10.2165/00003495-199651020-00006 8808167

[bb26] Yamaguchi, T., Hashizume, T., Matsuda, M., Sakashita, M., Fujii, T., Sekine, Y., Nakashima, M. & Uematsu, T. (1994). *Arzneim.-Forsch.* **44**, 59–64. 7907873

